# Current capabilities of large language models as peer reviewers for manuscripts submitted to ophthalmology-related journals

**DOI:** 10.1097/MD.0000000000048147

**Published:** 2026-03-27

**Authors:** Majid Moshirfar, Kenneth D. Han, Muhammed A. Jaafar, Mina M. Sitto, Manogna Nuthi, Kayvon A. Moin, Phillip C. Hoopes

**Affiliations:** a Hoopes Moshirfar Research Center, Hoopes Vision, Draper, UT; b Department of Ophthalmology, John A. Moran Eye Center, University of Utah School of Medicine, Salt Lake City, UT; c Utah Lions Eye Bank, Murray, UT; d University of Arizona College of Medicine-Phoenix, Phoenix, AZ; e Wayne State University School of Medicine, Detroit, MI; f Midwestern University College of Osteopathic Medicine, Glendale, AZ; g Department of Ophthalmology, Nassau University Medical Center, East Meadow, NY.

**Keywords:** artificial intelligence, ChatGPT, grading system, LLM, machine learning, peer review, rejection

## Abstract

To compare large language models (LLMs) and human reviewers in the peer review process of manuscripts submitted to 3 ophthalmology-related journals. This retrospective study comprised 300 randomly selected manuscripts from 3 anonymized journals under 1 editor between June 2023 and July 2024. Comments from 2 LLMs (Chat Generative Pre-Trained Transformer [ChatGPT] 4o and Gemini) and human reviewers (324 ophthalmologists) were compared. LLMs were prompted to accept, accept with major or minor revisions, or reject each manuscript in addition to providing comments. A 5-point Likert scale was used to assess the “favorability” of comments and compare manuscripts that were accepted or rejected by the editor. A 4-category quality assessment was used to compare the number of comments, detail/specificity, critical analysis, and literature support. Human reviewers rejected manuscripts more frequently (73.33% vs 2.00% ChatGPT and 2.00% Gemini; *P* < .001) and suggested major (22.67% vs 68.00% ChatGPT and 31.33% Gemini; *P* < .001) or minor revisions (3.33% vs 30.00% ChatGPT and 66.33% Gemini; *P* < .001) less often. Human reviewers gave more negative feedback for rejected manuscripts (−1.05 vs −0.02 ChatGPT and 0.24 Gemini; *P* < .015). ChatGPT repeated “novelty,” “sample size,” and “clarity” in 75%, 60%, and 50% of cases, respectively, while Gemini did so in 80%, 70%, and 65% of cases. Both lacked specificity, omitting line numbers and references. Although it is hoped that LLMs will one day be able to augment the role of peer reviewers, in their current state, LLMs should not be used for manuscript revision.

## 1. Introduction

The peer review process is intended to scrutinize and improve scientific papers shared between different physicians and scientists to promote innovative findings and advance the growing body of scientific literature as a whole.^[1]^ For many years, peer review has represented the gold standard for manuscript revision; as in many cases, it incorporates multiple perspectives from experts in a field and provides a thorough evaluation of a manuscript’s quality and the value that it might add to the current literature.^[1]^

However, due to the growing demands and workload faced by peer reviewers, some have suggested that the peer review process represents the “weakest link” in the medical research process.^[2]^ This can be attributed to peer review depending heavily upon personal integrity, diligence, and, in some subspecialties, the expertise of only a handful of true experts.^[3]^ In addition, there are increasing expectations of having an efficient peer review process with the goal of a quick publication.^[4]^ These limitations may be encountered to an even greater extent by smaller, less prestigious journals, which may not be able to attract as many reviewers. Because of these growing demands, there has been more interest in the use of automated tools such as large language models (LLMs) that can serve to augment the ability of peer reviewers to provide quality reviews for an even greater number of manuscripts.^[5]^

Given the recent rise in popularity of artificial intelligence (AI), it is no surprise that many have begun to wonder what role AI might play in scientific writing. Two of the most popular AI models currently include Chat Generative Pre-Trained Transformer (ChatGPT) 4o (OpenAI, San Francisco) and Gemini 1.5 Flash (Google, Mountain View). These have proven to be powerful tools when used carefully, and extensive research has been conducted assessing the utility of AI in performing literature searches, analyzing data, and writing manuscripts.^[6–12]^ Some studies have found that AI was able to help expedite these tasks, but frequently made errors in this process.^[13,14]^

However, while many studies have evaluated the performance of AI in producing scientific literature,^[6–11]^ few articles currently exist that compare its efficacy in reviewing manuscripts for publication with that of professional peer reviewers.^[3,15–17]^ The objective of our study is to explore the use of ChatGPT and Gemini in the peer review process of manuscripts submitted to 3 different ophthalmology-related journals.

## 2. Methods

### 2.1. Study design

This retrospective study involved 2 independent observers (P.C.H. and K.A.M.), both with backgrounds in ophthalmology, who collated 300 randomly selected manuscripts submitted to 3 ophthalmology journals within a single subspecialty and managed by 1 editor (M.M.), between June 2023 and July 2024. A single editorial account was used to facilitate standardized access and uniform data collection from journals with rigorous peer review processes and acceptance rates of approximately 20% to 25%. The 300 manuscripts included a total of 705 peer reviews by 324 ophthalmologists. Any studies categorized as a desk rejection or without any reviewers were excluded. Manuscripts were anonymized to avoid bias with respect to contributing authors and institutions. Specifically, the title page, author affiliations, and any mention of potentially identifying information were removed from each manuscript prior to entry into any AI model. Due to the preliminary nature of this study, the authors chose not to disclose the names of these journals to prevent any conclusions being drawn about the peer review process. The authors opted not to allow each AI platform to store and use information regarding manuscripts for training or other purposes to ensure the security of the data. None of the information provided to these AI models was saved to prevent future use. The peer review process was performed within a secure, on-premises enclave to protect the privacy of the input data. The study was approved by the Hoopes Vision Ethics Committee in Draper, Utah.

### 2.2. AI models

The AI models that were used in the study included ChatGPT-4o and Gemini 1.5. These 2 platforms were selected due to their popularity and ability to read PDF files, which enabled a comprehensive review process through the analysis of a variety of different types of manuscripts, graphs, figures, and tables. The control group used in this study consisted of human reviewers, specifically ophthalmologists. ChatGPT, Gemini, and these human reviewers are often referred to as the “3 groups” throughout this study.

### 2.3. Prompt design

The following prompt was developed to elicit feedback as well as 1 of 4 decisions from the AI models: “You are an invited journal reviewer tasked to review manuscripts for publication. Can you provide major positive or negative comments regarding this manuscript and whether you would accept, accept with minor revisions, accept with major revisions, or reject this paper in its current state?” This prompt, along with the PDF file of each of the 300 manuscripts, was sequentially input into new conversations with ChatGPT and Gemini, respectively. The frequency at which human reviewers, ChatGPT, and Gemini responded with each 1 of these 4 decisions was recorded and compared. Additionally, the most common phrases used during the review process by ChatGPT and Gemini were recorded.

### 2.4. “Favorability” assessment

To objectively assess the connotations of the comments provided by the 3 groups, whether positive or negative, the 2 observers (P.C.H. and K.A.M.) assessed the overall “favorability” of these comments. To do this, the observers used a 5-point Likert scale ranging from −2 to +2, where −2 represented “very negative comments,” 0 represented “equivocal comments,” and +2 represented “very positive comments” (Table 1). As a majority of manuscripts had multiple human reviewers, the observers assigned a favorability score based on a gestalt left by the human reviewers for each manuscript. For example, if 1 manuscript had 3 human reviewers who left comments with favorability scores of −2, −2, and +1, respectively, the observers would give an overall favorability score of −1 to the manuscript to capture the fact that the reviews were “mostly negative.” Then, these results were stratified based on the editor’s final decision of acceptance or rejection, and the favorability scores of each of the 3 groups were compared accordingly. To avoid bias, the observers were blinded to the source of the comments – whether they were produced by human reviewers, ChatGPT, or Gemini.

**Table 1:** Grading system for comment favorability.

Score	Description
−2	Very negative comments
−1	Mostly negative comments
0	Equivocal comments
1	Predominantly positive comments
2	Very positive comments

A 5-point grading scale (−2 to 2) was used to evaluate the comment favorability of human reviewers, ChatGPT, and Gemini.

ChatGPT = Chat Generative Pre-Trained Transformer.

### 2.5. Quality assessment

Additionally, the observers aimed to evaluate the quality of comments generated by the 3 groups. A 4-category quality assessment was implemented to evaluate the number of comments, detail/specificity, critical analysis, and literature support, with 0 being the worst and 3 being the best (Table 2). This grading system was adapted and modified based on previous works.^[16]^ A sample of 200 of the 300 studies was randomly selected for evaluation using a random number generator (Google Random Number Generator, Mountain View).

**Table 2:** Grading system for comment quality.

Category	Score	Description
Number of comments	0	No comments
	1	1–2 comments
	2	3–8 comments
	3	>9 comments
Detail/specificity	0	Made no references to any specific in-text sentences/phrases
	1	Mentioned a section of the paper, but did not specify line number
	2	Provided specific line numbers, but did not provide feedback
	3	Provided specific line numbers, feedback, and/or references to add to depth of paper
Critical analysis	0	Did not mention anything about results section or figures
	1	Mentioned overall general feedback on section, but did not go into specific comments
	2	Provided specific feedback on results/data
	3	Both specific feedback and suggestions to improve results/data
Literature support	0	Did not provide any references or support in argument of their comments
	1	Discussed other literature, but did not provide an example
	2	Referenced specific paper
	3	Provided a specific quote or segment from a reference

A 4-category grading scale (0–3) was used to assess the number of comments, detail/specificity, critical analysis, and literature support of comments from human reviewers, ChatGPT 4o, and Gemini. A score of 0 represents the lowest quality, and a score of 3 represents the highest quality in each category.

ChatGPT = Chat Generative Pre-Trained Transformer.

### 2.6. Statistical analysis

Microsoft Excel (version 16.0; Microsoft Corporation, Redmond), IBM Statistical Package for the Social Sciences (SPSS version 29.0; SPSS Inc., Chicago), and G*Power (version 3.1; Düsseldorf, Germany) were used for all data collection and statistical analysis.

Differences in the scores assigned by each observer to comments generated by human reviewers, ChatGPT, and Gemini were compared using paired *t* tests. No statistically significant differences were found between the ratings of the 2 observers. Thus, scores from the 2 observers were averaged. Additionally, the Kolmogorov–Smirnov test was used to analyze the normality of the data distribution for both favorability and quality assessment scores. As the data were not normally distributed for both comment favorability and quality, analysis of variance (ANOVA) tests were performed as they are considered sufficient for moderate violations of the normality assumption.

A chi-square test was used to compare the frequencies of the initial decisions by the 3 groups as to whether to accept, accept with major revisions, accept with minor revisions, or reject a manuscript for publication. Statistical significance was set at an α level of 0.015 according to the Bonferroni correction.

After stratifying by whether a manuscript was ultimately accepted or rejected by the journal, a one-way ANOVA with Tukey post hoc test was performed to evaluate any statistically significant differences in comment favorability scores between the 3 groups. Statistical significance was set at an α level of 0.015 according to the Bonferroni correction. The same analysis was performed to assess for differences in the review quality scores between human reviewers and the AI programs, with the same *P* value.

The statistical power of the one-way ANOVA was assessed using G*Power software. The effect size was calculated using Cohen *f*. For our Bonferroni-corrected α value of 0.015, a conservatively low effect size of 0.25, and 3 groups, a sample size of 210 was needed to achieve a statistical power of 0.80. Furthermore, post hoc analysis of our one-way ANOVA with a sample size of 300 reviews revealed a statistical power of 0.93.

## 3. Results

**Figure 1.**
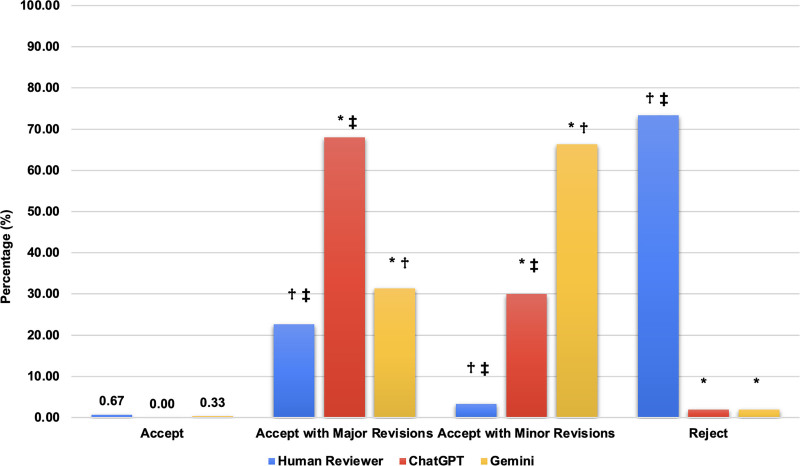
Comparison of the 4 decisions made by human reviewers, ChatGPT, and Gemini. Initial reviewer decisions of whether to accept, accept with major revisions, accept with minor revisions, or reject manuscripts made by human reviewers, ChatGPT, and Gemini, respectively. Notably, none of the 3 groups selected “Accept” as their initial response to a manuscript. A one-way ANOVA with Tukey post hoc test was performed with statistical significance set at *P* < .015. *Indicates significant compared with human reviewers. †Indicates significant compared with ChatGPT. ‡Indicates significant compared with Gemini. ANOVA = analysis of variance, ChatGPT = Chat Generative Pre-Trained Transformer.

A total of 300 manuscripts and 705 reviews were analyzed, resulting in an average of 2.39 (range: 1–7) human reviews per manuscript included in this study. Our analysis of the initial decisions made by human reviewers, ChatGPT, and Gemini revealed significant differences. Human reviewers initially chose to “Accept with Major Revisions” (22.67% vs 68.00% and 31.33%; *P* < .001) and “Accept with Minor Revisions” (3.33% vs 30.00% and 66.33%; *P* < .001) less frequently than both ChatGPT and Gemini, respectively (Fig. 1). Additionally, human reviewers chose to “Reject” manuscripts more frequently than both ChatGPT and Gemini (73.33% vs 2.00% and 2.00%; *P* < .001). Additionally, ChatGPT chose to “Accept with Major Revisions” more frequently than Gemini (68.00% vs 31.33%; *P* < .001) and chose to “Accept with Minor Revisions” less frequently than Gemini (30.00% vs 66.33%; *P* < .001). Notably, the 3 groups rarely provided the initial decision of “Accept” (0.67% vs 0.00% vs 0.33%; *P* > .015).

**Figure 2.**
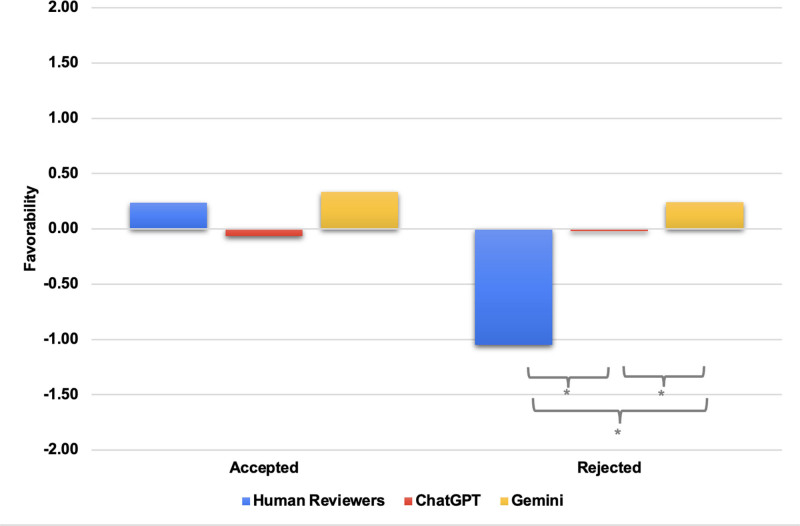
Comparison of comment favorability scores between human reviewers, ChatGPT, and Gemini. Comment favorability of human reviewers, ChatGPT 4o, and Gemini stratified by manuscripts that were accepted or rejected by the journal. A score of −2 represents the most negative comments, and a score of +2 represents the most positive comments. A one-way ANOVA with Tukey post hoc test was performed with statistical significance set at *P* < .015. *Indicates a statistical significance of *P* < .015. ANOVA = analysis of variance, ChatGPT = Chat Generative Pre-Trained Transformer.

After stratifying by whether the assigned editor (M.M.) ultimately chose to accept or reject a manuscript, human reviewers were found to provide similar positive comments compared with ChatGPT and Gemini for manuscripts that were accepted (0.24 vs −0.06 vs 0.33; *P* > .015) (Fig. 2). However, for manuscripts that were rejected by the editor, human reviewers provided more negative comments than both ChatGPT and Gemini (−1.05 vs −0.02 vs 0.24; *P* < .015). Additionally, ChatGPT provided more negative comments compared with Gemini for the manuscripts that were ultimately rejected by the editor (−0.02 vs 0.24; *P* < .015).

**Figure 3.**
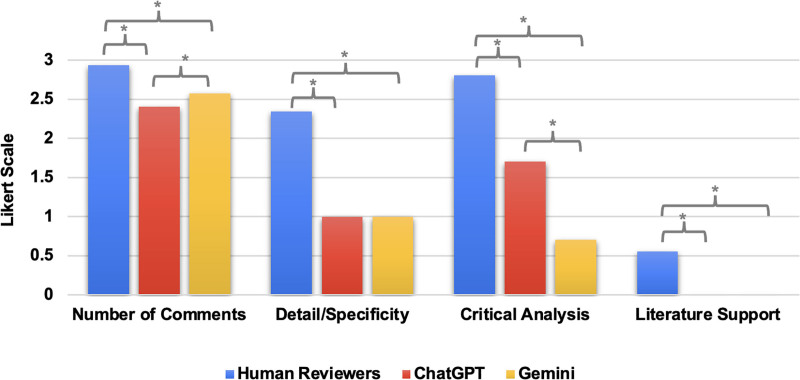
Comparison of comment quality scores between human reviewers, ChatGPT, and Gemini. Four-category quality assessment of comments from human reviewers, ChatGPT, and Gemini, comparing the number of comments, detail/specificity, critical analysis, and literature support. A score of 0 represents the lowest quality, and a score of 3 represents the highest quality in each category. *Indicates a statistical significance of *P* < .015. ChatGPT = Chat Generative Pre-Trained Transformer.

According to the 4-category quality assessment created to compare reviewer quality, human reviewers outperformed ChatGPT and Gemini to a statistically significant degree in terms of the number of comments (human reviewer 2.94, ChatGPT = 2.40, Gemini = 2.58; *P* < .001), detail/specificity of reviews (human reviewer = 2.35, ChatGPT = 1.00, Gemini = 1.00; *P* < .001), critical analysis (2.81 vs 1.71 vs 0.71; *P* < .001), and literature support (0.55 vs 0.00 vs 0.00; *P* < .001). Additionally, when comparing the quality of comments provided by each of the AI models, Gemini provided a greater number of comments than ChatGPT (ChatGPT = 2.40, Gemini = 2.58; *P* < .001) but performed more poorly in terms of critical analysis (ChatGPT = 1.71, Gemini = 0.71; *P* < .001) (Fig. 3).

Additionally, the AI models were found to repeat the same comments for many manuscripts. Of the 300 manuscripts analyzed, the most redundant verbiage included “novelty,” “sample size,” and “clarity.” For instance, ChatGPT 4o mentioned “novelty,” “sample size,” and “clarity” in 75%, 60%, and 50% of manuscripts reviewed, respectively. Gemini mentioned “clarity,” “novelty,” and “sample size” in 80%, 70%, and 65% of the manuscripts reviewed, respectively. This trend was not observed among human reviewers.

Qualitatively, both AI models provided approximately 6 to 9 comments per manuscript, whereas human reviewers left a variable number of comments, ranging from around 1 to 16. However, neither AI model provided specific line numbers, references, or statistical tests as suggestions for a single manuscript, while human reviewers provided this type of feedback frequently.

## 4. Discussion

There has been an exponential rise in the use of AI in the scientific community, attributable to its speed and efficiency. However, only a few studies have been performed assessing the ability of AI to serve as a manuscript reviewer.^[3,15–17]^ Additionally, these previous studies were published in the fields of diabetes and metabolic syndrome, as well as in computer methods, biomedical programs, and biology. Our study investigated the use of AI in the process of reviewing scientific manuscripts in comparison with professional human reviewers in the field of ophthalmology.

In general, this study found that AI models tend to demonstrate a reluctance to provide definitive decisions of accepting or rejecting manuscripts, preferring instead to respond with “Accept with Major Revisions” or “Accept with Minor Revisions” (Fig. 1). This may represent a feature of their design that prevents them from making strong recommendations one way or another, which may pose liability issues in certain situations.^[18]^ However, this creates a significant limitation of these AI models in manuscript review, which is a process that requires confident decision-making abilities,^[19]^ especially when faced with a far greater number of manuscripts than would be able to be published by 1 academic journal.

This is further demonstrated through the analysis of feedback provided regarding manuscripts that were ultimately accepted and rejected by the journal. Human reviewer comments were more consistent with the final decision by the journal, as they appropriately provided more positive and negative comments for articles that were accepted and rejected, respectively. Conversely, both AI platforms failed to align with the judgment capability of humans, grading all manuscripts similarly. Suleiman et al had similar findings to our study as they demonstrated the lack of congruence in comments between human reviewers and AI across 20 articles.^[16]^ Interestingly, Saad et al found that responses by ChatGPT were mostly positive and ChatGPT was unable to identify papers that failed to meet editorial standards.^[15]^ These conclusions support our study’s findings that AI in its current state lacks the ability to assess manuscripts appropriately and to provide reliable feedback.

ChatGPT and Gemini performed poorly in terms of critical analysis compared with human reviewers. In contrast, Biswas et al found that ChatGPT was able to critically analyze manuscripts in a manner that was comparable with human reviewers. However, the use of an extremely small sample size (n = 1) in their study may have prevented the authors from noticing the pattern of AI models repeating the same generic feedback, which may be mistaken for critical analysis. As mentioned earlier, we found that both ChatGPT and Gemini repeatedly used verbiage such as “novelty,” “sample size,” and “clarity” throughout the majority of their manuscript reviews, often simply pulling these terms from the limitations section of the manuscript being analyzed.

In addition, the human reviewer’s knowledge in the specific field of ophthalmology was shown to be invaluable in many instances, as it enabled them to provide references to help guide revisions and assess novelty. Throughout our study, neither AI platform provided a specific reference, highlighting the lack of analytical reasoning and understanding of the current body of literature possessed by the human reviewers.^[20,21]^ Previous studies have found that AI models were able to provide literature support, but most of the references used included inaccurate information or were completely fabricated.^[14,22]^ This further emphasizes the detailed human knowledge required in the peer review process, which current AI platforms lack.

Additional specific examples of limitations to AI reviewer feedback are as follows: 1 manuscript was well written but ultimately rejected by the human reviewers because it focused on basic science research that was felt to be better suited for a different journal. However, AI was not able to recognize this discrepancy in topic and recommended accepting this article. In another instance, a proposed surgical technique was rejected by human reviewers largely due to unnecessary costs with unclear advantages over the gold standard. Again, AI did not seem to have an awareness of this potential financial cost and recommended accepting the article. Finally, it was noted that 1 manuscript received negative feedback from multiple human reviewers for failing to adhere to the conventional case report format. However, AI did not recognize this and actually stated that the article was well formatted. These are all instances of human reviewers’ knowledge and expertise improving the peer review process and adding layers of detail orientation and specificity that the current AI platforms lack.^[17,20,21]^

While this study demonstrated that human reviewers are far superior in the peer review process than AI in its current state, there are some significant limitations to human reviewers that must also be recognized. Depending on the journal, human reviewers are sometimes able to see the names, institutions, and countries from which authors are submitting manuscripts. This may introduce biases that AI platforms do not have. Additionally, while AI consistently provides a sufficient quantity of comments, human reviewers at times leave an insufficient number of comments, which may consist of only a few sentences. However, despite these flaws, human reviewers remain the gold standard for the peer review process.

This study has several limitations. First, the analysis was restricted to manuscripts from 3 journals within a subspecialty of ophthalmology assigned to a single editor and included only 2 popular LLMs. These factors may affect the external validity of this study, as editorial practices and journal selectivity may vary across editors and journals. Second, although all manuscripts were within a single ophthalmology subspecialty, studies were not further classified by design or subject area, which may also limit the generalizability of this study. Additionally, the assumption of independence required for ANOVA may have been violated because multiple reviews were nested within individual manuscripts, potentially affecting statistical inference of the results. Furthermore, while structured grading systems were used to minimize subjectivity, they could still benefit from further refinement and validity verification. Finally, AI models may perform better with a greater emphasis on prompt engineering; however, this was not the focus of our paper, as human reviewers typically provide feedback without such detailed instructions. For example, perhaps prompting LLMs to provide specific line numbers would have improved the specificity of comments. However, this study is the first of its kind in the field of ophthalmology regarding the peer review process, featuring a robust sample size of 300 manuscripts, over 700 reviews from 324 ophthalmologists, and the incorporation of both quantitative and qualitative assessments. Further studies should be conducted with larger sample sizes, responses derived from a more diverse set of journals, and the inclusion of additional AI models.

## 5. Conclusion

This study evaluated the ability of 2 AI models, ChatGPT 4o and Gemini, to be able to augment the manuscript peer review process. These AI models were able to provide comments on a variety of different papers, but in their current states, more work is needed regarding the quality, detail, and literature support of these comments. Additionally, when it comes to deciding whether to accept or reject a manuscript, both AI models were found to be indecisive and unwilling to provide a strong recommendation. Given the current demanding nature of the peer review process, it is hoped that AI will one day be able to augment the ability of human reviewers to provide quality feedback to a greater number of manuscripts. In its current state, it can be concluded that AI should not be used in the review process, as it lacks deductive reasoning and is unable to provide a comprehensive and adequate analysis of scientific literature.

## Acknowledgments

During the preparation of this manuscript/study, the authors used ChatGPT 4o (OpenAI, San Francisco) and Gemini 1.5 Flash (Google, Mountain View) to compare their performance in the peer review process of ophthalmology journals.

## Author contributions

**Conceptualization:** Majid Moshirfar, Phillip C. Hoopes.

**Investigation:** Majid Moshirfar, Kenneth D. Han, Muhammed A. Jaafar, Mina M. Sitto, Kayvon A. Moin, Phillip C. Hoopes.

**Methodology:** Majid Moshirfar, Kenneth D. Han, Muhammed A. Jaafar, Mina M. Sitto, Manogna Nuthi, Kayvon A. Moin.

**Project administration:** Majid Moshirfar, Phillip C. Hoopes.

**Supervision:** Majid Moshirfar, Mina M. Sitto, Kayvon A. Moin, Phillip C. Hoopes.

**Validation:** Majid Moshirfar, Kenneth D. Han, Muhammed A. Jaafar, Mina M. Sitto, Manogna Nuthi, Kayvon A. Moin.

**Writing – original draft:** Majid Moshirfar, Kenneth D. Han, Muhammed A. Jaafar, Mina M. Sitto, Manogna Nuthi, Kayvon A. Moin, Phillip C. Hoopes.

**Writing – review & editing:** Majid Moshirfar, Kenneth D. Han, Muhammed A. Jaafar, Mina M. Sitto, Manogna Nuthi, Kayvon A. Moin, Phillip C. Hoopes.

**Formal analysis:** Kenneth D. Han, Muhammed A. Jaafar.

**Software:** Kenneth D. Han, Muhammed A. Jaafar.

**Visualization:** Kenneth D. Han, Muhammed A. Jaafar, Mina M. Sitto, Manogna Nuthi, Kayvon A. Moin.

**Data curation:** Manogna Nuthi.
